# The application of dual-heart nursing mode in patients with coronary heart disease and angina pectoris and its impact on anxiety, depression, and quality of life

**DOI:** 10.1097/MD.0000000000036530

**Published:** 2023-12-29

**Authors:** Lu Li, Piaopiao Tan, Gaoya Li, Shengxiang Yang, Meng Guo, Cangyun Zhang

**Affiliations:** a Department of Cardiology, The Central Hospital of Enshi Tujia and Miao Autonomous Prefecture, Enshi, Hubei, China.

**Keywords:** coronary heart disease angina pectoris, double heart care, Hamilton depression scale, quality of life

## Abstract

To diagnose and treat patients with coronary heart disease and angina pectoris with dual heart care mode and analyze the treatment effect. Three hundred cases meeting the inclusion criteria were equally divided into 3 groups, each containing 50 male and female cases. The patients in experimental group 1 took the dual heart nursing method proposed by the subject; experimental group 2 received betastatins; control group received conventional treatment. After 12 weeks of treatment, Hamilton depression scale scored the 3 groups, and their anxiety and depression scores, clinical manifestations, symptom scores and self-acceptance were analyzed. The chi square value of these data was compared with *P*, and judge whether they meet the needs and differences of statistical data. Then compare their scores before and after treatment to identify the treatment status. The anxiety and depression scores of experimental group 1 were the lowest among the 3 groups, with the values of 59.62 ± 7.925 and 58.64 ± 6.416; The total patients who responded effectively to treatment in experimental group 1 accounted for 83%, and the patients who responded effectively to treatment rate was the highest in the 3 groups; The effect of decreasing the score of complications in experimental group 1 was also the most obvious, from 9.07 ± 4.28 to 3.14 ± 2.07, which was the best in the 3 groups; the self-evaluation of patients in experimental group 1 was the highest among the 3 groups, 89.72 ± 4.28. The proposed dual heart care and treatment method can effectively treat coronary heart disease and angina pectoris, and can effectively improve the clinical performance and self-acceptance of patients. It can effectively restore the anxiety and depression of patients after treatment, and then improve patients’ life quality, which has the value of popularization and use.

## 1. Introduction

For the elderly, cardiovascular disease can not only harm their health, but also produce many negative emotions in their hearts.^[1]^ Patients with cardiovascular disease often have anxiety, depression and other emotions, which will bring great inconvenience to their treatment.^[2–4]^ Among these diseases, the disease of coronary heart disease, due to the hardening of the coronary artery in the heart, causes symptoms such as insufficient blood supply to the myocardium and cardiac hypoxia, which in turn leads to myocardial ischemia and cardiac convulsion. The primary manifestation of this condition upon onset is angina pectoris, which is the major contributing factor to both physical and mental exhaustion in cardiovascular patients.^[5–7]^ Therefore, while treating angina pectoris, greater attention should also be paid to the psychological issues it causes. However, currently, the primary targeted treatment method in major hospitals is statin therapy. Nevertheless, this approach requires the use of expensive drugs and cannot achieve holistic treatment of both the physical and mental aspects.^[8]^ This not only places a greater financial burden on patients but also does not yield complete treatment efficacy in all cases. This suggests that a more efficient new treatment approach still needs to be explored.

Therefore, this study aims to investigate whether dual cardiac care therapy is more effective in treating this condition through comparative research. The goal is to achieve better results, addressing not only angina but also the negative psychological emotions in patients, such as anxiety and depression, in a mutually reinforcing manner, thereby enhancing and promoting the overall treatment effectiveness.

## 2. Materials and methods

### 2.1. General information

The study was supported by the Ethics Committee of Enshi Central Hospital (2023 No. 23). Patients with coronary heart disease and angina pectoris were selected from the Cardiovascular Center of the Central Hospital of Enshi Tujia and Miao Autonomous Prefecture from January to December 2022. The subject confirmed that these patients presented with different degrees of anxiety and depression symptoms, which had an impact on their lives. Three hundred patients with coronary heart disease from January to December 2022 were selected. Based on different treatment methods, patients were randomly assigned to 3 groups, experimental group 1 (E1), experimental group 2 (E2), and control group (C), using simple randomization, with each group consisting of 100 cases.^[9]^ E1 received double heart nursing method for 12 weeks; E2 received Betastatins for 12 weeks; C received conventional treatment for 12 weeks, and they were scored with Hamilton Depression Scale. The 3 groups of patients contained 50 male and 50 female cases. E1 aged from 66 to 85 years old with an average of 64.82 ± 9.07, most of which were between 65 and 75 years old; E1 aged from 62 to 87 years old with an average of 65.07 ± 9.42, widely belonging to 68 to 72 years old; E1 aged from 66 to 82 years old with average age of 66.15 ± 9.36, mostly distributed in 70 to 78 years old. Bio-Ethical Committee has approved this project, and family members of older patients have signed the informed consent.

Inclusion criteria: ① Aged 60 to 90 years (including 60 and 90 years). ② The patient was diagnosed through electrocardiography, cardiovascular angiography, and MRI. ③ Patients are full of depression or anxiety about life, and their quality of life is low. ④ Patients can communicate normally, know and consent to the subject. ⑤ The Hamilton Depression Scale score of the patient was above 10. ⑥ The patient’s education level was high school or above. Exclusion criteria: ① the depression and anxiety in patients were not caused by angina pectoris but rather by severe psychiatric symptoms that met the criteria for a psychiatric diagnosis. ② Patients who completely lose confidence in life or are unwilling to cooperate with this project. ③ Patients who cheat in filling out the questionnaire. ④ Patients with myocardial infarction or severe heart failure confirmed by experts and physicians. ⑤ Patients who cannot tolerate the dual heart nursing treatment mode. ⑥ Patients aged <60 or >90. ⑦ Pregnant or lactating patients. Patients with severe disease of other major organs.

**Figure 1.**
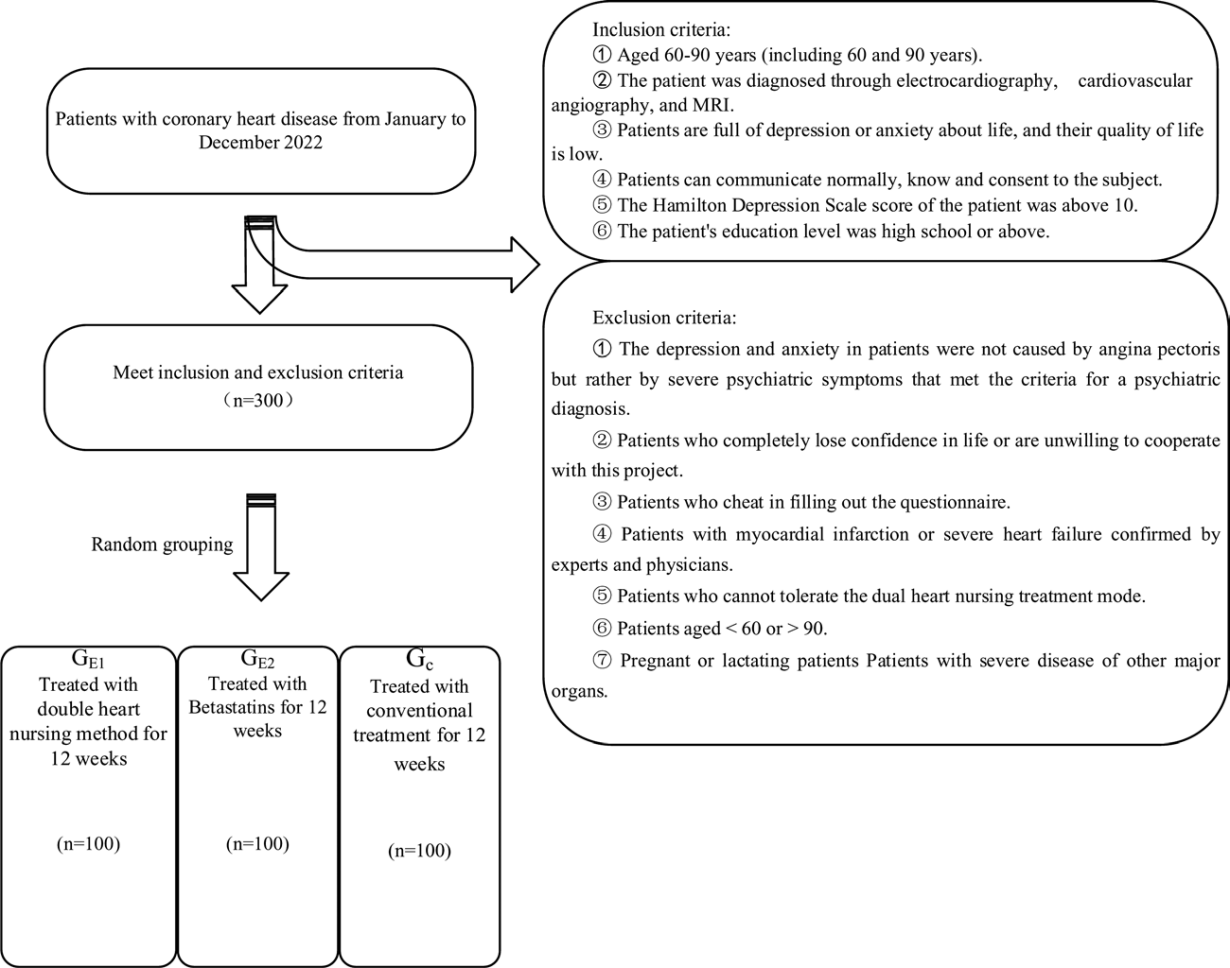
Patient group flow chart.

Shedding criteria: ① patients who are not treated according to the prescribed treatment methods. ② Patients with serious complications during treatment (Fig. 1).

### 2.2. Diagnosis and treatment methods

Experimental scheme: in this project, E1 received 2 heart nursing method for 12 weeks; E2 received were treated with Betastatins for 12 weeks; C received conventional treatment for 12 weeks, and they were scored with Hamilton Depression Scale.^[10–12]^ Two heart nursing treatment method: 100 patients in experimental group 1 assembled at 2:00 PM each day, and the assembly location was determined based on the situation, with open locations being selected.; The nursing doctor on duty will choose the video of cardiac rehabilitation dance to guide the patient’s movement while playing it. When the patient is performing activities, a professional is required to be present to ensure that the patient’s physical indicators are good, and there are no symptoms such as chest pain and tightness. When the gymnastics time reaches 35 to 55 minutes, the nurse will concentrate the patients and lead them to a quiet place. The environment shall effectively avoid flash, noise and activities of irrelevant personnel; Patients should be well dressed, emotionally stable, and empty their gastrointestinal tract. At this time, the nurse began to play videos of stable myocardial function and guide the patient to perform correct actions. This activity should be supervised by the chief physician in real time to avoid abnormal conditions of patients.^[13]^ Precautions during diagnosis and treatment: before treatment, dual heart nursing should be approved by the nursing and ethics department, and accompanied by the head nurse or the director of Cardiology Department; Nurses in the Department of cardiology should be trained in psychological knowledge and pass the examination of cardiac knowledge. During the time of the project, patients and their family members have the right to know the purpose and significance of the project, and they have agreed and cooperated. Patients in the betastatins group were administered betastatins orally at a daily dose of 10 mg, taken in the evening. The control group received traditional treatment, primarily using nitroglycerin for symptomatic relief, along with lifestyle modifications, including smoking cessation and a low-fat diet. For Hamilton Depression Scale, some patients with high knowledge can fill in independently. When patients have questions about it, the accompanying nursing staff should answer them in time.^[14–16]^

### 2.3. Observation index

Safety observation indexes: cardiac function indexes of patients; blood routine, chest X-ray and MRI indexes; adverse reaction indicators. Efficacy observation index: the symptom changes of the cases before and after treatment were recorded according to Hamilton Depression Scale. Responded effectively index of coronary heart disease: the ECG score of the patient is higher than 80 points, and the degree of self-acceptance is more than 85 points. Long term treatment: the Hamilton Depression Scale score will be given to the patients half a year after the end of treatment. Thrombus index: to assess the formation of blood clots in the patient’s bloodstream by measuring the level of D-dimer in their blood.

### 2.4. Efficacy evaluation criteria

Responded effectively to treatment standard: the patient’s symptoms of cardiac pain basically disappear, or will not affect routine activities, and the use of cardiac drugs will be stopped. The patient’s Hamilton Depression Scale score was lower than 10 points, no longer had depression and anxiety, and the quality of life was improved. Effective standard: Although the patient still has periodic cardiac pain, the routine activities can still be carried out as usual, and the use of cardiac drugs is reduced by more than half. The Hamilton Depression Scale score of the patients was lower than 10, the mood gradually tended to be positive, and the quality of life improved. Invalid criteria: the patient’s ECG has not changed, which affects the patient’s routine activities, and the normal use or increment of cardiac drugs. And it will have an impact on daily life. The Hamilton Depression Scale score of the patient is higher than 10 points, and the mood is still depressed and anxious. The responded standard of long-term curative effect: the Hamilton Depression Scale score of patients with coronary heart disease and angina pectoris who responded effectively to treatment 6 months after treatment was lower than 10 points. Recurrence criteria for long-term efficacy: the patients who responded effectively to treatment were rescored with Hamilton Depression Scale 6 months after the end of treatment, and the score was higher than 10 points.

### 2.5. Statistical methods

This topic uses EpiData3.2 to establish a data set and test the experimental results on both sides, and then uses SPSS22.00 software for statistical analysis. Chi-square analysis is used to compare differences in data composition. Paired *t*-tests are employed for within-group comparisons of pre- and posttreatment scores. Intergroup *t*-tests are used to compare the magnitude of differences between groups. The exact probability was determined by the mean ± standard deviation and chi square. When the subject setting is *P* < .05 or 0.01, the data is statistically significant.

## 3. Results

### 3.1. Analysis of general information of patients

Three groups of patients, 300 in total, were introduced. Among them, the male to female ratio of patients selected by the project is the same, and the age distribution is basically the same. Illness duration ranged from 0 to 12 months, and the patients in each time group had little difference. Table 1 shows the analysis results of general data in this project.

**Table 1:** Analysis of general data results.

Gender	Male		Female		Chi square value	*P*
Experimental group (1) Experimental group (2)	48 46		49 50		0.656	.305
Control group	49		49			
Age (years)	60–66	67–73	74–80	81–87	Chi square value	*P*
Experimental group (1) Experimental group (2)	4 12	55 58	12 15	26 11	−0.568	.578
Control group	22	35	27	14		
Course of disease (Months)	0-3	3-6	6–12	>12	Chi square value	*P*
Experimental group (1) Experimental group (2)	25 28	27 26	24 20	21 22	−0.728	.602
Control group	29	25	31	13		

It can be seen from Table 1 that patients in the 3 groups all fell off. Among them, there were 3 cases of exfoliation patients in the experimental group 1, 4 cases of exfoliation in E2, and 2 cases of exfoliation in C. The analysis results in Table 1 showed no difference in the distribution of gender, age and duration of illness among the 3 groups.

### 3.2. Self-assessment scores of anxiety and depression of patients before and after treatment

This topic compared the Hamilton Depression Scale scores of patients before and after treatment to judge the treatment effect. The self-rating Anxiety Scale (SAS) and self-rating Depression Scale were used to comprehensively evaluate the psychological status of patients. The subject prepared 600 copies of these 2 kinds of test papers, respectively for patients before and after treatment. The subject investigated the 3 groups of patients, and a total of 1192 test papers were recovered. A total of 1180 valid test papers were recovered after excluding invalid test papers such as missing filling, and their average scores are shown below. We first applied the chi-square test to compare the percentage difference of patients with scores >10 before and after treatment (Table 2).

**Table 2:** Hamilton Depression Scale scores of the 3 groups before and after treatment.

Time interval	Before treatment		After treatment		Chi square value	*P*
Index	SAS	SDS	SAS	SDS	/	/
Experimental group (1) Experimental group (2)	65 71	68 69	42 65	45 62	5.288 5.127	.005 .003
Control group	76	72	74	72	4.095	.001

**Figure 2.**
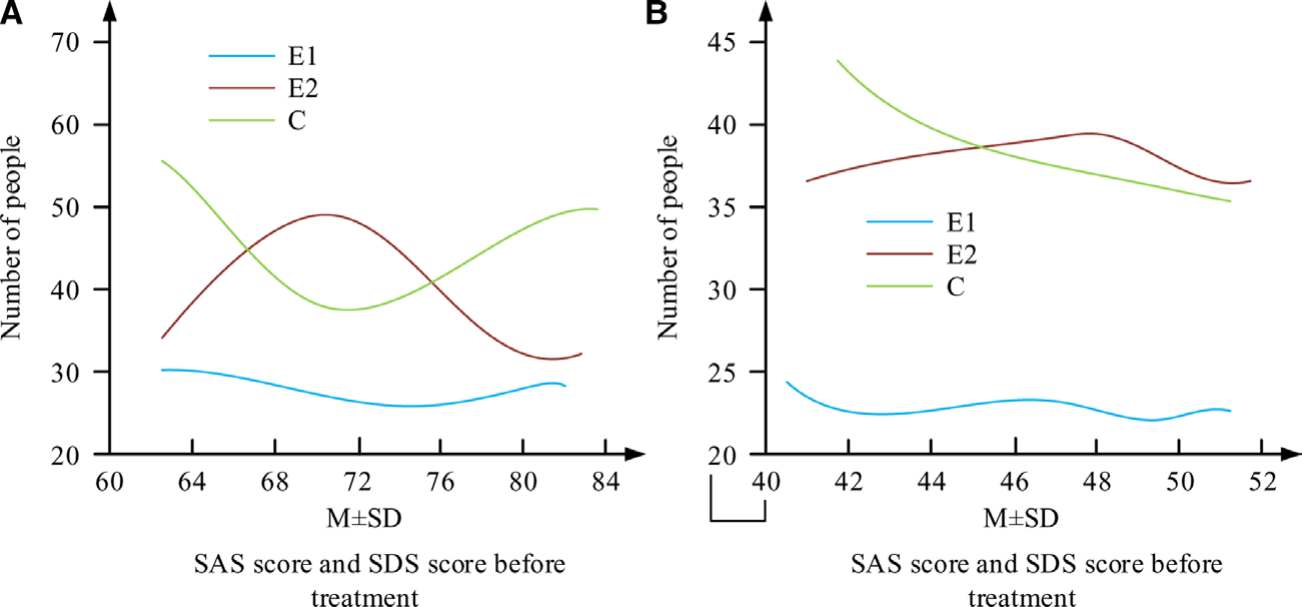
The relationship between the average SAS and SDS scores and the standard deviation of 3 groups.

Table 2 shows that SAS and SDS of experimental group 1 decreased effectively after treatment; although the 2 values of experimental group 2 did not decrease significantly, they were still better than C’s experimental results. E1 chi square value was 5.236, which was the best in the 3 groups, and the chi square values of the other 2 groups were 5.288 and 4.095, respectively. The *P* of the control group was 0.001. That of 2 experimental groups were 0.002 and 0.005, indicating that 3 groups’ scores were not only statistically significant (*P* < .05), but also different. So the subject plotted the relationship between the mean and standard deviation of SAS and SDS scores of the 3 groups, as shown in Figure 2.

It can be seen from Figure 2A that before treatment, the relationship between the mean and standard deviation of SAS and SDS scores of experimental group 1 was 59.62 ± 7.925 and 58.64 ± 6.416, respectively; The relationship in experimental group 2 was 60.56 ± 8.074 and 59.41 ± 6.728, respectively; The relationship in the control group was 61.44 ± 8.625 and 60.87 ± 7.014, respectively. In Figure 2B, after treatment, the relationship of experimental group 1 was 52.48 ± 7.074 and 48.52 ± 6.188, respectively; the relationship in experimental group 2 was 60.47 ± 8.004 and 57.65 ± 6.614, respectively; the relationship in the control group was 61.39 ± 8.623 and 60.72 ± 7.011, respectively. The specific comparative analysis results are presented in Table 3. Table 3 indicates that paired t-tests were conducted to compare the scores before and after treatment within each group. According to the results with P < .05, it can be observed that all 3 groups showed improvements in depressive symptoms among coronary heart disease patients. Table 4 represents that based on paired data before and after treatment, intergroup *t*-tests were performed. The results with *P* < .05 indicate differences in treatment score improvements among the 3 groups. According to the average values, it can be seen that experimental group 1 (E1) had a larger decrease in SAS and SDS scores, followed by experimental group 2 (E2). This indirectly demonstrates the superior efficacy of the dual cardiac therapy.

**Table 3:** Paired *t*-tests within treatment groups for pre- and posttreatment score comparisons.

Time interval	Before treatment		After treatment		T	*P*
Index	SAS	SDS	SAS	SDS	/	/
Experimental group (1) Experimental group (2)	59.62 ± 7.925 60.56 ± 8.074	58.64 ± 6.416 59.41 ± 6.728	52.48 ± 7.074 60.47 ± 8.004	48.52 ± 6.188 57.65 ± 6.614	5.42 5.36	.004 .002
Control group	61.44 ± 8.625	60.87 ± 7.014	61.39 ± 8.623	60.72 ± 7.011	4.45	.003

**Table 4:** Comparison of the change magnitude in the Hamilton Depression Rating Scale among the 3 groups.

Time interval	The difference	
Index	SAS	SDS
Experimental group (1) Experimental group (2)	7 ± 2.1 1 ± 3.2	10 ± 2.2 2 ± 1.1
Control group	0.5 ± 0.4	0.3 ± 0.6
*P*	<.001	.002

### 3.3. Comparison of clinical manifestations of patients before and after treatment

Among the patients treated in this project, 48% were significantly effective in the treatment of coronary heart disease and angina pectoris, and 32% were effective. The proportion of patients with ineffective treatment was 12%, and the proportion of patients who responded effectively to treatment and relapsed patients with long-term curative effect was 2% and 3%, respectively. The total patients who responded effectively to treatment accounted for 83%, and the total ineffective responded patients accounted for 14%. In the experimental group 2, 25% of the patients were significantly effective in disease treatment, and 24% were effective in treatment. Patients with ineffective treatment accounted for 42%, patients with long-term curative effect and patients with recurrence accounted for 1% and 2%, respectively. The total effective responded patients accounted for 50%, and the total ineffective responded patients accounted for 44%. In the control group, 12% patients were significantly effective in disease treatment, and 15% patients were effective in treatment. The patients who failed to respond to treatment accounted for 53%, and the responded patients and relapsed patients with long-term efficacy accounted for 4% and 12%, respectively. The total effective responded patients accounted for 31%, and the total ineffective responded patients accounted for 65%. The responded ratio of E2 and C was relatively low, and the comparison between the proportions of the 3 groups of patients is shown below.

**Figure 3.**
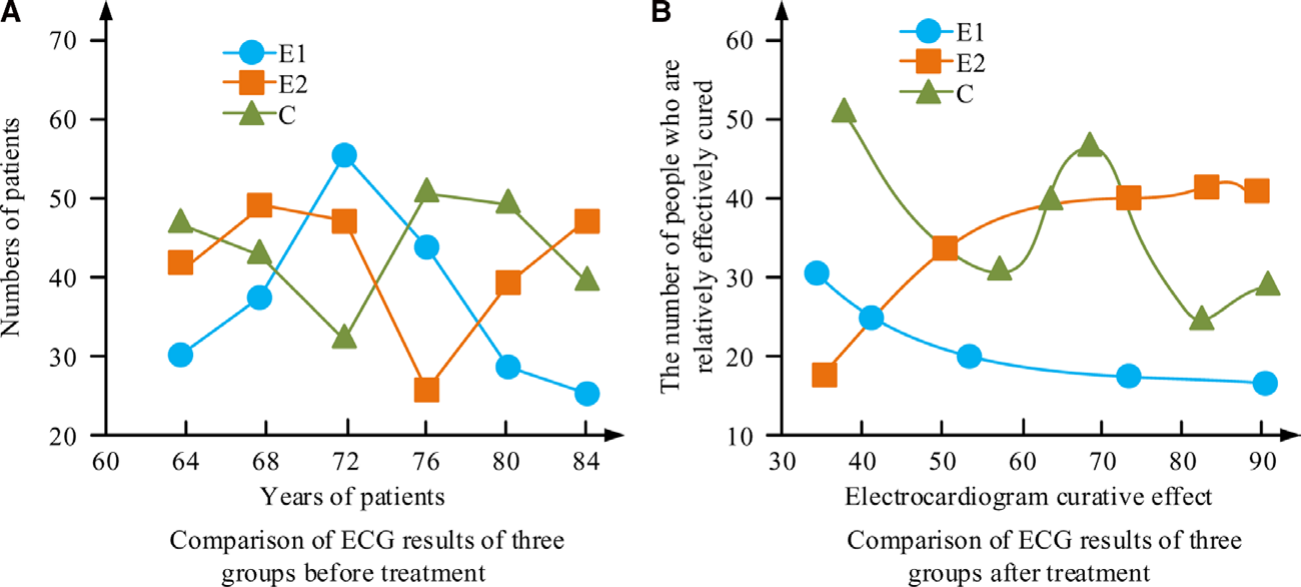
Comparison of ECG between 3 groups of patients before and after treatment.

According to Table 5, the responded rate of the experimental group 1 was the highest, 83%, and the responded rates of the other 2 groups were 50% and 31%, respectively. And *P* < .05 in the 3 groups, these data are statistically significant. It shows that under the same conditions, the dual heart care therapy proposed in this topic is more effective for coronary heart disease and angina pectoris. In order to verify the treatment effect of the patient, the subject compared the ECG of the patient before and after treatment, and drew the result figure as shown in Figure 3.

**Table 5:** Comparison of the proportion of effective and ineffective treatment among 3 groups of patients.

Index	Effective treatment	Treatment usefully	Ineffective treatment	Long term responded	Long term recurrence	t	*P*
Experimental group (1) Experimental group (2)	48% 25%	32% 24%	12% 42%	2% 1%	3% 2%	7.012 6.944	.003 .004
Control group	12%	15%	53%	4%	12%	6.451	.004

*Note*: Chi square value is 10.763, *P* < .05.

Figure 3 shows ECG effects comparison of 3 groups. It can be seen from Figure 2 that the average ECG quality of experimental group 1 is the highest, 87.5. 72.7, and 61.4 in the other 2 groups, respectively. It shows that in the study of responded patients’ ECG, the recurrence rate of patients in experimental group 2 and control group is higher.

### 3.4. Score comparison of patients’ symptoms before and after treatment

**Figure 4.**
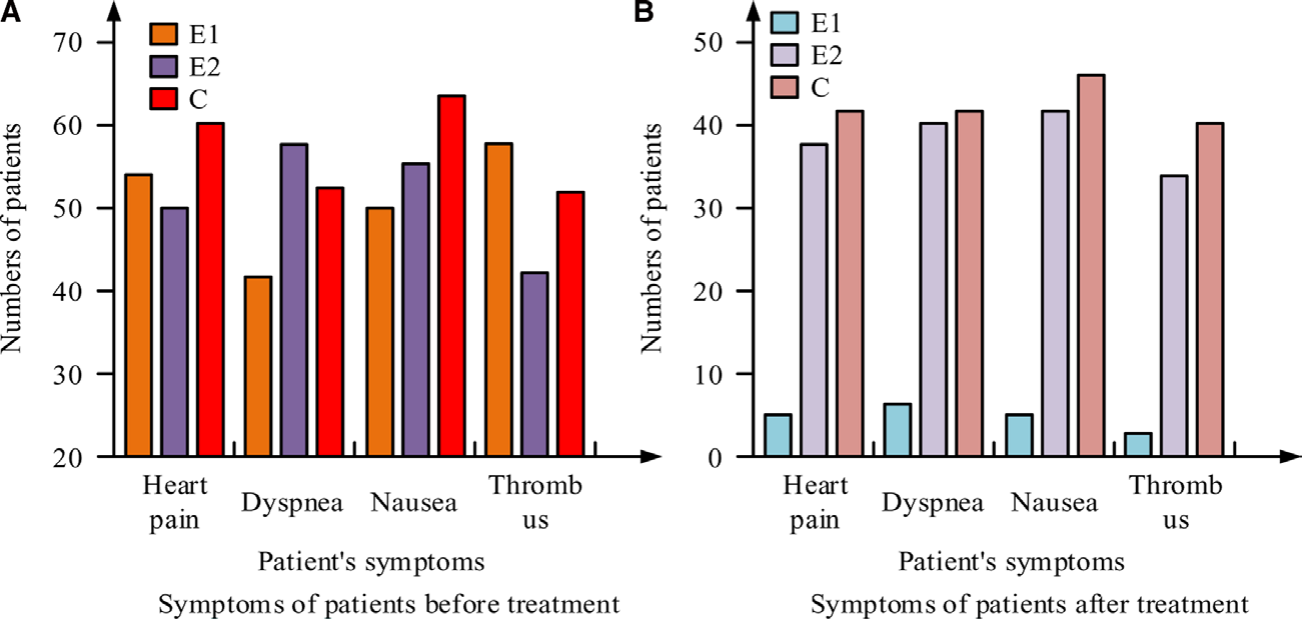
Comparison of the scores of 4 symptoms before and after treatment.

For patients, the symptoms of angina pectoris of coronary heart disease include cardiac pain, dyspnea, dizziness, nausea, and thrombosis. This project compares the scores of patients before and after treatment for these 4 symptoms, and the comparison results are shown in Figure 4.

Figure 4 shows the score comparison results of patients. It can be seen from Figure 4 that before treatment, the complications of patients with coronary heart disease and angina pectoris were compatible, that is, there were multiple complications in the same patient. However, the number of patients with complications in E1 was reduced to <10 after treatment with the treatment method proposed by the project, and the average reduction rate was 84%. The average reduction rate of patients in E2 and C was 52% and 36%, respectively. It shows that the patients with coronary heart disease and angina pectoris have the best recovery of complications after dual heart care. The integral of these data was calculate and the record was obtained as shown in Table 6.

**Table 6:** Four kinds of complications of patients before and after treatment.

Integration	Number of cases	Total symptom score		t	*P*
		Before treatment	After treatment	/	/
Experimental group (1)	96	9.07 ± 4.28	3.14 ± 2.07*^#^	7.016	<.01
Experimental group (2)	97	9.36 ± 5.69	4.07 ± 2.15*^#^	6.953	<.05
Control group	93	9.43 ± 4.75	4.55 ± 4.54*	6.874	<.05

* Indicates the comparison within the integral group before treatment, *P* < .01.

# Indicates the comparison between the score groups after treatment, *P* < .01.

Table 6 shows that patients in experimental group 1 had the most obvious effect of reducing the score of complications after dual heart treatment, from 9.07 ± 4.28 before treatment to 3.14 ± 2.07 after treatment. While the patients in the other 2 groups were 4.07 ± 2.15 and 4.55 ± 4.54 respectively after treatment, indicating that this method has good effect for patients with coronary heart disease and angina pectoris. The chi square values of the 3 groups were 7.016, 6.953, and 6.874, respectively, and the value of experimental group 1 was the highest, and *P* < .01 of the 3 groups of patients, indicating that these data were statistically significant.

### 3.5. Comparison of patients’ self-acceptance before and after treatment

The research shows that the degree of acceptance of patients after treatment is related to the treatment effect. This project is aimed at the patients in 3 groups to study their own acceptance scores after treatment. The subject selected the Hamilton Depression Scale score, and then collected these data, as shown in Table 7.

**Table 7:** Patient’s self-acceptance score.

Group	Body score	Cardiac stability score	Disease cognitive score	Aggregate score
Experimental group (1)	32 ± 4.04	35 ± 4.17	22 ± 3.97	89.72 ± 4.28
Experimental group (2)	25 ± 4.10	21 ± 4.15	24 ± 2.19	67.65 ± 3.85
Control group	13 ± 2.12	9 ± 4.06	25 ± 3.85	52.83 ± 3.20
t	8.829	7.034	6.685	/
*P*	<.01	<.01	<.01	/

**Figure 5.**
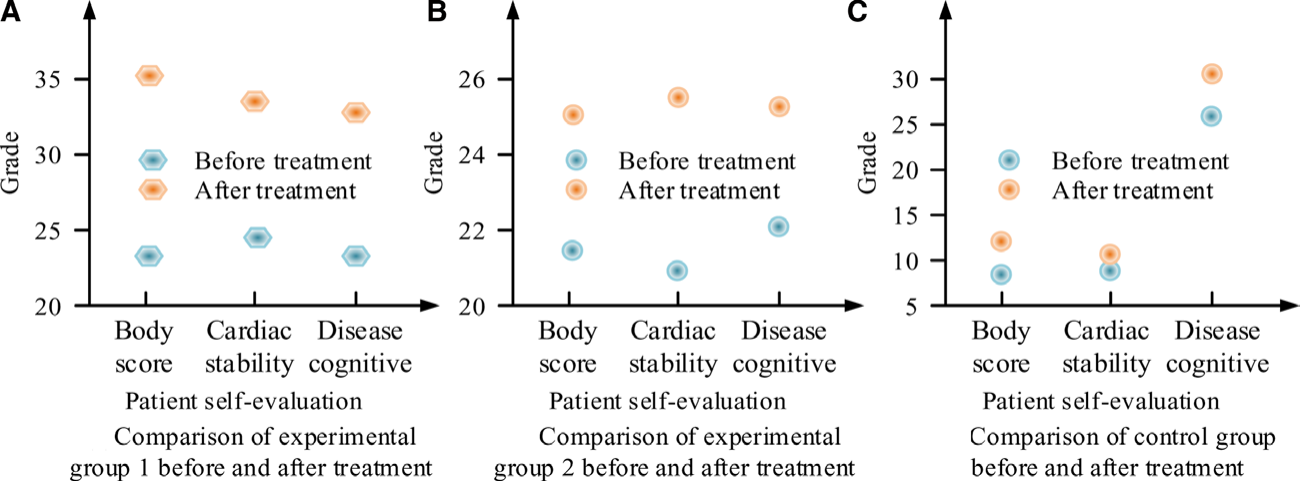
Data comparison between 3 groups before and after treatment.

Table 7 shows that after treatment, patients in experimental group 1 had the highest self-evaluation among the 3 groups, including patients’ scores on their own condition, morphological stability and pathological characteristics. The P < .01 of the 3 data shows that they are statistically significant, and there are differences in the data. However, the data after treatment is not comprehensive enough. In order to verify the accuracy of dual heart care, this project compared the data before and after treatment, and obtained the results shown in Figure 5.

It can be seen from Figure 5 that for patients in the 3 groups, the degree of self-acceptance showed an upward trend after treatment, while the scores in the 3 groups before treatment were low. Among them, the change effect of experimental group 1 was the most obvious, 25 ± 1.75, and the change values of the other 2 groups were 13 ± 2.08 and 5 ± 3.64, respectively. It shows that the psychological recovery of patients with coronary heart disease and angina pectoris is the best after the proposed dual heart care treatment.

## 4. Discussion

Angina pectoris of coronary heart disease is a common disease of the elderly. It not only makes patients in a painful state at the onset, but also increases patients’ negative emotions, which can affect their life quality.^[17–20]^ In order to improve the impact of this disease, 300 patients aged 60 to 90 consisted of 3 groups according to the patient data.^[21–23]^ The subject selected the dual heart nursing mode to treat the patients, and compared the SAS and SDS scores, clinical manifestations, symptom scores and self-acceptance before and after treatment.^[24,25]^ The analysis was conducted on SAS and SDS scores. Initial comparison of the percentage of patients with Hamilton scores >10 before and after treatment revealed that experiment group 1 had the most significant reduction in SAS and SDS scores after treatment, with an χ² value of 5.236, which was the highest among the 3 groups. The χ² values for E2 and C were 5.288 and 4.095, respectively. The corresponding *P*-values for the 3 groups were 0.001, 0.002, and 0.005, indicating that the SAS and SDS scores in all 3 groups had both statistical significance (*P* < .05) and differences. Subsequently, paired *t*-tests were performed to compare the differences in Hamilton scores before and after treatment within each group, revealing statistically significant differences. Furthermore, the scores in all 3 groups decreased after treatment, demonstrating that all 3 experimental groups had a certain effect on improving the patients’ symptoms. We then compared the magnitude of score reduction among the 3 groups, further confirming that experiment group 1 had the most significant reduction in SDS scores, with values of 7 ± 2.1 and 10 ± 2.2, which was statistically significant. These results indicate that dual-heart care treatment in experiment group 1 is superior to traditional treatment in Group 3 and vascular treatment alone in group 2 in improving depression and anxiety associated with coronary heart disease.

In the clinical data of patients, the total effective responded patients accounted for 83% in the experimental group 1, and the total ineffective responded patients accounted for 14%; In experimental group 2, 50% of the patients were effectively responded, and 44% of the patients were ineffective; In C, the total effective responded patients accounted for 31%, and the total ineffective responded patients accounted for 65%. It shows that the responded rate of experimental group 1 is the highest. For the ECG analysis of the subject, the average ECG quality of 3 groups were 87.5, 72.7 and 61.4, respectively. It shows that in the study of patients who responded effectively to treatment, the experimental group 1 has the best curative effect, while the other 2 groups have the risk of recurrence. The patient’s symptoms include cardiac pain, dyspnea, dizziness, nausea and thrombosis, so this topic compares the patient’s score before and after treatment for these 4 symptoms. Patients with angina pectoris of the same coronary heart disease may have a variety of complications. After the treatment of experimental group 1, the number of patients with complications decreased to <10, and the average reduction rate was 84%. The average reduction rate of patients in E2 and C was 52% and 36%, respectively. It shows that the patients with coronary heart disease and angina pectoris have the best recovery of complications after dual heart care. Patients in experimental group 1 also had the most obvious effect of reducing the score of complications after double heart treatment, from 9.07 ± 4.28 before treatment to 3.14 ± 2.07 after treatment. While the patients in the other 2 groups were 4.07 ± 2.15 and 4.55 ± 4.54 respectively after treatment, indicating that this method has good effect for patients with coronary heart disease and angina pectoris. The chi square values of the 3 groups were 7.016, 6.953, and 6.874, respectively, and the value of experimental group 1 was the highest, and *P* < .01 of the 3 groups of patients, indicating that these data were statistically significant.^[26–28]^

In addition, this project also aimed at the patients in the 3 groups, and studied their own acceptance scores after treatment, including patients’ scores on their own condition, morphological stability and pathological characteristics. The self-evaluation of patients in experimental group 1 was the highest among the 3 groups, 89.72 ± 4.28, 67.65 ± 3.85, and 52.83 ± 3.20 in E2 and C, respectively. The *P* < .01 of 3 data shows that they are statistically significant, and there are differences in the data. To verify the accuracy of double heart nursing, the data before and after treatment were compared.^[29,30]^ For the patients in the 3 groups, the degree of self-acceptance showed an upward trend after treatment. Among them, the change effect of experimental group 1 was the most obvious, 25 ± 1.75, and the change values of the other 2 groups were 13 ± 2.08 and 5 ± 3.64, respectively. It shows that the psychological recovery of patients with coronary heart disease and angina pectoris is the best after the proposed dual heart care treatment.

In conclusion, dual-heart care therapy can provide a more comprehensive improvement in both the psychological and physical aspects of coronary heart disease compared to previous treatment with vascular medications alone. It enhances the psychological well-being of patients and improves their overall quality of life. This study offers a treatment option for coronary heart disease patients, especially those with comorbid depression and anxiety, providing a more holistic approach to healthcare that aims to address both physical and psychological aspects, ultimately achieving better outcomes.

There are still some limitations to this study. Firstly, this project was conducted exclusively on elderly patients. Investigating the effectiveness of this approach in younger patients with coronary heart disease and angina is equally important. Therefore, as this research project further develops, this aspect will be addressed in future studies. Secondly, this study only provides an initial assessment of the treatment efficacy of dual-heart care therapy and does not delve into the underlying mechanisms. Further in-depth mechanistic research will be conducted in subsequent studies.

## Author contributions

**Conceptualization:** Lu Li, Piaopiao Tan, Gaoya Li, Shengxiang Yang, Cangyun Zhang.

**Data curation:** Lu Li, Piaopiao Tan, Cangyun Zhang.

**Formal analysis:** Lu Li, Cangyun Zhang.

**Investigation:** Lu Li, Piaopiao Tan, Gaoya Li, Shengxiang Yang, Meng Guo, Cangyun Zhang.

**Methodology:** Lu Li, Piaopiao Tan, Gaoya Li, Shengxiang Yang, Meng Guo, Cangyun Zhang.

**Supervision:** Piaopiao Tan, Shengxiang Yang, Meng Guo, Cangyun Zhang.

**Validation:** Shengxiang Yang.

**Visualization:** Piaopiao Tan, Gaoya Li, Meng Guo.

**Writing – original draft:** Lu Li.

**Writing – review & editing:** Lu Li, Cangyun Zhang.

## References

[1] Tripathi P Sarma MS Yachha S. Gastrointestinal polyps and polyposis in children: experience of endoscopic and surgical outcomes. Dig Dis. 2021;39:25–32. 32450557 10.1159/000508866

[2] Armstrong PW Pieske B Anstrom KJ. Vericiguat in patients with heart failure and reduced ejection fraction. N Engl J Med. 2020;382:1883–93. 32222134 10.1056/NEJMoa1915928

[3] Hassan R Ahmed SB. Sex differences in heart failure and precision medicine: right patient, right time… wrong dose? Heart. 2021;107:1692–3. 34407967 10.1136/heartjnl-2021-319831

[4] Wang Y Lu W Chen H. Analysis of the mechanism of Huatan Tongluo Formula (HTTLF) combined with western medicine in the treatment of chronic heart failure complicated with depression based on molecular docking and co-precipitation technology. J Biol Regul Homeost Agents. 2022;36:2105–12.

[5] Yang M Liu J. Comments on “Traditional Chinese medicine use in the treatment of acute heart failure in western medicine hospitals in China: analysis from the China PEACE retrospective heart failure study”. Chin J Integr Med. 2020;26:163–4. 31776961 10.1007/s11655-019-2713-7

[6] Zhao L Sheng YY Xue YZ. A systematic review and meta-analysis of Dengzhanxixin injection for coronary heart disease with angina pectoris patients. TMR Integr Med. 2023;7:e23007–10.

[7] Ying C Xue X Xiaolin X. Traditional Chinese medicine in the prevention and treatment of stable angina pectoris in patients with coronary heart disease based on the theory of. J Tradit Chin Med. 2021;41:150–6. 33522208 10.19852/j.cnki.jtcm.2021.01.017

[8] Wang ZX Ban JF Li RL. Network-meta analysis of 9 kinds of patent Chinese medicine for nourishing qi and activating blood in the treatment of angina pectoris of coronary heart disease. J Hainan Med Coll. 2021;27:37–37.

[9] Perera D Berry C Hoole SP. Invasive coronary physiology in patients with angina and non-obstructive coronary artery disease: a consensus document from the coronary microvascular dysfunction workstream of the British Heart Foundation/National Institute for Health Research Partnership. Heart. 2023;109:88–95. 10.1136/heartjnl-2021-320718 PMC9811089 35318254

[10] Oslund S Washington C So A. Multiview robust adversarial stickers for arbitrary objects in the physical world. J Comput Cogn Eng. 2022;1:152–8.

[11] Liu Y Li Z Wang X. Effects of adjuvant Chinese patent medicine therapy on major adverse cardiovascular events in patients with coronary heart disease angina pectoris: a population-based retrospective cohort study. Acupunct Herb Med. 2022;2:109–17.

[12] Li HZ Qin W Zhang YY. Onset of coronary heart disease is associated with HCMV infection and increased CD14+ CD16+ monocytes in a population of Weifang, China. Biomed Environ Sci. 2020;33:573–82. 32933609 10.3967/bes2020.076

[13] Li J Cao GY Zhang XF. Chinese medicine She-Xiang-Xin-Tong-Ning, containing moschus, corydalis and ginseng, protects from myocardial ischemia injury via angiogenesis. Am J Chin Med. 2020;48:107–26. 31931593 10.1142/S0192415X20500068

[14] Sharma JB Deora S Choudhary R. Diagnostic utility of mitral annular displacement by speckle tracking echocardiography in predicting significant coronary artery disease in suspected chronic stable angina pectoris. Echocardiogr. 2020;37:2010–7. 10.1111/echo.14900 33131121

[15] Jia-ming SU Jing P Hai-min C. Identification of genes related to tubulointerstitial injury in type 2 diabetic nephropathy based on bioinformatics and machine learning. J Hainan Med Coll. 2022;28:43–51.

[16] Tian CY Huang Y Sun X. The efficacy and safety of clopidogrel and aspirin in coronary heart disease with angina pectoris: a systematic review and meta-analysis. Asian Toxicol Res. 2020;2:97–108.

[17] Gao SW Liu ZC Wang ZX. Meta analysis of Xuesaitong injection in the treatment of angina pectoris of coronary heart disease. J Hainan Med Coll. 2021;27:37–45.

[18] Zhong L Zhou XY. Application progress of cognitive behavioral therapy in coronary heart disease. TMR Integr Nurs. 2021;5:160–2.

[19] Lazović N Milićević A Trifunović-Zamaklar D. Clinical characteristics and two-year outcome of patients with angina pectoris without obstructive coronary artery disease: diabetics vs non-diabetics. Medicinski Podmladak. 2020;71:13–20.

[20] Li F Ji Z Du R. Influence of integrated traditional Chinese and Western medicine nursing on the living quality of patients with angina pectoris under the concept of evidence based nursing. Indian J Pharm Sci. 2021;83:231–6.

[21] Ni ZT Shao ZB. Clinical efficacy of angina pectoris after pci in patients with coronary heart disease complicated with type 2 diabetes by Yiqi Yangyin and Huatan Tongluo recipe. J Hainan Med Coll. 2020;26:35–9.

[22] Bozaci I Ozkan O Ozkan A. The relationship between serum vaspin levels and the degree of coronary involvement in patients with stable angina pectoris Stabil angina pektorisli hastalarda serum vaspin düzeyleri ile damar tutulum derecesi arasindaki ilişki. Haseki Tip Bulteni. 2020;58:176–82.

[23] Mihajlović D Maksimović M Dojčinović B. Acute coronary syndrome (STEMI, NSTEMI and unstable angina pectoris) and risk factors, similarities and differences. Scripta Medica. 2020;51:252–60.

[24] Zhang H Lei P Zhang S. Research progress of gualou xiebai in the treatment of coronary heart disease. Proc Anticancer Res. 2021;5:86–90.

[25] YuL S Zajniddinov FA Borshchev GG. The results of complex surgical treatment of patients with coronary heart disease. Clin Med. 2020;98:766–71.

[26] Rui-han LI Jian-zhen Z Kai-lin H. Efficacy of Wendan decoction series for coronary heart disease with anxiety and depression: a systematic review and meta-analysis. J Hainan Med Coll. 2022;28:40–9.

[27] Bjørnestad E Olset H Dhar I. Circulating trimethyllysine and risk of acute myocardial infarction in patients with suspected stable coronary heart disease. J Intern Med. 2020;288:446–56. 32270523 10.1111/joim.13067

[28] Li X Mo X Liu T. Efficacy of *Lycium barbarum* polysaccharide in adolescents with subthreshold depression: interim analysis of a randomized controlled study. Neural Regener Res. 2022;17:1582–7. 10.4103/1673-5374.330618 PMC8771081 34916444

[29] Zhang F Wang F Li CH. Therapeutic effects of subthalamic nucleus deep brain stimulation on anxiety and depression in Parkinson’s disease patients. J Neurorestoratology. 2022;10:31–42.

[30] Lu YY Lu XM Shao CY. Empathetic nursing with mindful cognitive therapy for fatigue, depression, and negative emotions in leukemia patients undergoing long-term chemotherapy. World J Clin Cases. 2022;10:1826–33. 35317141 10.12998/wjcc.v10.i6.1826 PMC8891779

